# Supplementary material for: Household antibiotic use in Malawi: A cross-sectional survey from urban and peri-urban Blantyre
Source: PLOS Glob Public Health. 2023 Aug 11;3(8):e0001946. doi: 10.1371/journal.pgph.0001946 (PMC10420364; doi:10.1371/journal.pgph.0001946)
Supplement: Attachment — Submitted filename: response to reviewers.pdf [file pgph.0001946.s001.pdf]

Reviewer 1

Comment #1.1: Thank you very much for the opportunity to review this manuscript. Unregulated antibiotics use by the community in low- and middle-income countries is a major factor in the development of AMR. The cross-sectional survey reported in this manuscript adds to the body of knowledge in this subject area and I would like to thank the authors for this work.

Author's Response: Thank you very much for the positive comments. We are pleased that you feel the manuscript provides an important contribution to the literature.

Comment #1.2: What informed the selection of the study sites? How did you deal with possible selection bias?

Author's Response: Thank you for the comment. We purposively selected these sites for several reasons. Firstly, for the two urban sites (Chilomoni and Ndirande), we had geospatially resolved household data from a previous cluster randomised trial to allow us to use this as a sampling frame, supporting random sampling from the community and minimising selection bias. Secondly, having conducted many large community-based trials and surveys in Blantyre over the past decade, we are very familiar with the socio-economic conditions within these neighbourhoods, and so are confident that they are representative of Blantyre as a whole and generalisable to other urban centres in Southern and Eastern African countries. Thirdly, with strong community relations within these neighbourhoods, through previous quantitative and social science work, we were able to undertake sensitive interviewing techniques to minimise response bias. We recognise that the two urban sites were densely populated; to provide a contrast we selected a peri-urban site (Chileka) to ensure that our estimates could be more broadly applicable to peri-urban as well as urban settings. Table 1 Shows that there were differences in characteristics between these neighbours and so we are assured that our selection criteria were valid. Finally, we were able to use a rigorous sampling approach within neighbourhoods to further minimise bias.

We have added a sentence to the discussion to note that there may be some residual heterogeneity in neighbourhoods outside our survey sites not captured by the sampling approach.

Lines: 311-315:

Neighbourhoods were purposively sampled on the basis of the availability of sampling frames from previous surveys and demographic/poverty profiles; we did however randomly sample of households within neighbourhoods, and this may limit generalisability.

Comment #1.3: Why were two neighbouring urban sites (Chilomoni and Ndirande) compared with one peri-urban site (Chileka)? For a better comparison of community antibiotic use in Malawi, the inclusion of a typical rural site could have been done (i.e., one each of urban, peri-urban and rural randomly selected).

Author's Response: Thanks for this. We have addressed some of these issues in point 1.2 above. Additionally, we were limited in our ability to recruit further sites by funding. Please see change where we have added text to address this point.

Lines: 311-315
Neighbourhoods were purposively sampled on the basis of availability of sampling frames from previous surveys and demographic/poverty profiles; we did however randomly sample households within neighbourhoods, and this may limit generalisability.

Comment #1.4: Table 1: Details of the Self-rated household poverty scales 2 – 5 are missing.

Author's Response: We apologise for this; we have added footnotes to the table to describe the scale. This self-rated household poverty scale has previously been extensively used in the Malawi National Integrated Household Surveys.

Lines: 162-164:
Self-rated household poverty was ascertained by asking respondents to rate their household's poverty status compared to their neighbours on one of 6 steps, with 1 being the poorest in the neighbourhood, and 6 being the wealthiest in the neighbourhood.

Comment #1.5: Under Antibiotic use: Lines 199 – 201 – The sentence "The bag 200 included multiple versions of the same antibiotics (active ingredients) to facilitate 201 recognition" is not a finding. It should have been included in the explanation of the "drug bag" in the Methods section.

Author's Response: Thanks we have moved this to the Methods.

Reviewer 2

Comment #2.1: Thanks for the opportunity to review this well-written and timely manuscript that addresses an important question in the development of AMR in low-and middle-income countries, and the impact of community-level antibiotic use. The manuscript has many strengths including its thorough sampling methodology, novel use of the drug-bag methodology, and statistical analysis. The manuscript highlights the important issue of both overuse and limited access to antimicrobials in these resource-poor contexts. Data of these kinds are severely lacking from contexts such as Malawi and therefore I would like to recommend this manuscript for publication with a few points that the authors may want to consider.

Malawi-Liverpool-Wellcome Trust Clinical Research Programme
P.O. Box 30096, Chichiri, Blantyre 3
Tel: +2651 1 81 24 23 / +2651 811 918 // Fax: +2651 875 774

Author's Response: Thanks for these helpful comments.

Comment #2.2: Did you collect data on where households were acquiring their antimicrobial? Studies from south-Asia demonstrate extensive use of private pharmacies without the need for formal healthcare consultation, which is important when it comes to regulation. Patterns of healthcare utilisation are quite different in southern/eastern Africa, and I think some comment on this in the discussion would be instructive.

Author's Response: Thanks, we agree this is important, however, we did not collect this information. We have added a sentence to the discussion highlighting this is a limitation and a priority for future research.

> Lines: 314-315:
>
> We did not collect data on where participants obtained antibiotics from; this should be a priority for future research projects and surveillance.

Comment #2.3: From published surveillance studies performed in Blantyre (e.g., Musicha et al, Lancet ID and Meiring et al, Lancet GH, there are others) it is striking how the commonest causes of bacterial infection are resistant to the antibiotics that people are using in their homes that you have highlighted through this study. The recognition of and use of ciprofloxacin is surprisingly low, and then amoxicillin with clavulanate, macrolides and 3rd generation cephalosporins are completely absent. I think it will strengthen your argument on lack of access to appropriate healthcare provision if you connect the dots between your findings and the results of these surveillance studies.

Author's Response: Thank you this is helpful. We have now added text to the Discussion to emphasis these points.

> Lines: 238-240:
>
> Previous studies in Malawi have shown rapidly emerging bacterial resistance to these antibiotics, threatening clinical and public health utility [24-25]

Reviewer 3

Comment #3.1: Include Malawi because the study is targeting Malawi. .

Author's Response: Thank you we have now added Malawi to the sentence.

Malawi-Liverpool-Wellcome Trust Clinical Research Programme
P.O. Box 30096, Chichiri, Blantyre 3
Tel: +2651 1 81 24 23 / +2651 811 918 // Fax: +2651 875 774

Lines: 5-6:
However, knowledge is limited about the ways antibiotics are used in communities in Malawi and sub-Saharan Africa.

Comment #3.2: WHO is appearing for the first time so write in full and put that in bracket

Author's Response: Thanks we have corrected this.

Comment #3.3: Where heterogeneous in this case shall mean? And there are examples coming up I guess to illustrate the heterogeneity, but there is no linkage to show that. 'For example' can help sorting out that issue. Both these studies' should be checked.

Author's Response: Thank you. We apologise, but we are not completely clear what the Reviewer means here.

Comment #3.4 This line and onwards shows that the study is narrowing down to self-medication. However the approach to that has no justification why the study focused on that area. It would be better to state a few of such bad practices and single out the 'self-medication' and state why.

Author's Response: Thanks for this observation. The study set out to capture all sources of antibiotic use and recognition, not just self-medication. We are unclear what the review means by "bad practice" here.

Comment #3.6: 53 The introduction goes back to general issues about antimicrobial use practices. So it's difficult to tell from the introduction about its focus. So either talk in general terms of the uses or narrow down to self-medication.

Author's Response: Thank you. We have carefully revised the introduction to ensure clarity of argument throughout.

Comment #3.7: 62 Is there any reason for choosing the 3 sites? Of all the areas in Malawi, Blantyre or urban areas? Of course line 78 tries to explain this, but I do not think the explanation makes it any different from other place or act as justification because the stated reasons are not unique to the areas mentioned, unless if there is data to back that up or if there is, the sentence to should rewritten to bring it out.

Author's Response: Thanks for these points, we have addressed these in Response 1.2 above.

Comment #3.8: In other parts, citations are put after a full stop and here it's before. So there must be consistency. One way and replicate throughout the document.

Author's Response: Thanks we have now corrected these.

Comment #3.9: 90 Which census is that? 2018 or 2008 census? How did you get the geo-referenced data?

Author's Response: The census is already described as being conducted in 2015 in this sentence. This was not the Malawi National Statics office census, but a census conducted citywide in Blantyre for a series of cluster randomised trials. We have now added references to these surveys.

Comment #3.10: 77 There is a mention of HIV prevalence. Is there any relationship of the study to it or it is just mentioned for the reader's attention? This is not clear although I have seen it coming out in the discussion section. If the HIV was the basis of the study, or selection of the study sites, it should have been stated.

Author's Response: Thanks for this comment. HIV prevalence is important because people living with HIV, have more frequent antibiotic use than people who are HIV negative, for example, for chemoprophylaxis and treatment of opportunistic infections. We have added text to this sentence to explain.

Lines: 240-243

In contexts such as Malawi, cotrimoxazole is commonly used for chemoprophylaxis for people living with HIV, and with HIV prevalence in the study populations being high, it is not surprising that this was one of the most commonly used and recognised antibiotics.

Comment #3.11: 101 How did you select the households? Did you select all those geo-referenced in the previous studies or selected some out of those? I expect that your sample sizes were different and yours might have been smaller than those earlier studies unless stated otherwise. How many people belonged to all the households and how many were not eligible due to either age or declined consent? Since you have mentioned that you involved all members of a household but few were left out.

Author's Response: Thanks we describe the sampling approach in lines 91-95. The sampling frame was all households within the neighbourhood, and we randomly selected 75 households in each neighourhood. We have now added a sentence to describe the sample size calculation which was conducted a priori. Sample

Table 1 shows the number of households we were able to recruit in each neighbourhood. We do not have data on the number that were excluded as this was not systematically collected.

> Lines: 130-133:
>
> Sample size estimates were based on a previous survey, which estimated that ~3% of participants reported using one antibiotic at the time of interview.[25] A sample size of 75 participants per neighbourhood gave a precision of +/- 3% around a point estimate of 3% antibiotic use within each neighbourhood.

Comment #3.12: 109-118 You have mentioned how you recruited the head of household, but not the other members in the households. How were the other members recruited? And how did you ensure that there is no response contamination from previous respondent of the household on the new respondents of the same family? And how did you ensure privacy of the respondents from other family members?

Author's Response: We asked the head of the household about the household composition and use. The interviews were conducted at the home of the respondent, and other members of the household were present and discussed responses. We knew in the design of the study it would be difficult to maintain privacy at the household level. We therefore did not ask participants to disclose if they were uncomfortable and the study team were trained to pick up on any discomfort and move on. We have added a line on this in the methods.

> Lines: 119-121:
>
> The primary respondent was the household head, however, other members of the family did provide answers during the pile-sorting exercise.

Comment #3.13: 118 How did you deal with the issue of repackaged antibiotics since some of them are not sold in their original packs? Are there no colour mix-ups between antibiotics of different APIs in Malawi that you would be sure that colour would be enough for someone to remember previous use of that medicine? In the comparisons of the neighborhood, how did you deal with the issue of different education backgrounds of the respondent's as well as levels of income? Because these factors can affect the comparative analysis. Or how did you deal with these confounders?

Author's Response:

We had limited statistical power for subgroup analysis, e.g. by educational status or income level. We agree that larger surveys should explore these potential confounders in greater detail. To assemble the drug bag, we visited both formal and informal drug sellers and included drugs from the government sector. This built on our experiences in the ethnographic research we undertook. We included multiple options for antibiotics and a range of packaging that allowed participants to interact with the drugs.

Comment #3.14: 175 Talking about household respondents, yet the population in the respondent's is 217. So were all the responses in a household counted as 1 or each was independent? Because if you talk about household respondents, I expect a population bigger than 217, because that's what it is in the text, 1051. Or are you giving the responses of household heads only this time?

Author's Response: Yes, these responses are at the household level.

Comment #3.15: Figure 2. Were there no capsules in the drug bag? Or the word tablets is being used interchangeably with capsules because mostly antibiotics are packed in capsules.

Author's Response: We used the term capsules interchangeably with the word tablets.

Comment #3.16: Antibiotics brands differ from place to place and period to period. How did you deal with that issue? Because the time of putting up a drug bag might differ from the time the respondents used a particular drug which might also affect its presence during the questioning. Was there a time limit to which the respondents recruited had used the drugs because the longer the time, the higher the chances of forgetting drug colour or product name especially when you don't anticipate and future use of such information.

Author's Response: We assembled the drug bag within 3 months of collating the antibiotic drug bags. We had previously trailed the method during ethnographic studies and found that the most frequently and recognised antibiotics were those dispensed from the public sector. The packaging, colour and shape of the drugs were quite uniform. However, we have noted this as a limitation of the study.

Lines 303-306
The drug bag uses a wide range of antibiotics available in the community; however it is possible that we did not include some important formulations, or that packaging designs have changed, limiting recognition.

Comment #3.17: 218 Were there any attempts to relate the suspension use to number of children in the households?

Author's Response: No we did not look at this. The sample size is likely too small for this sort of analysis

Comment #3.18: 230 There is an attempt to link cotrimoxazole to HIV, yet this drug is used for many diseases as well or is this the only indication for this drug in Malawi Treatment Guideline? And is it used strictly for that purpose in Malawi and that it can only be accessed in strict ways? How was this arrived at to be attributed to HIV? Were there any questions related to the reasons for using a particular antibiotic? Or were there any statistical tests that showed this possibility? If so, highlight them in the discussion by putting a table reference. Amoxicillin was also found to be used widely. Why is it also not attributed to the HIV? Dont Amoxicillin and Cotrimoxazole have similar uses in one way or the other? Take note that antibiotics are also widely known to be used in colds and flu apart from their intended uses.

Author's Response: Thank you for these helpful comments. We have previously investigated this in detail in our ethnographic work (MacPherson and MacPherson). International donor programmes did provide antibiotics for specific programmes such as HIV, TB and STI. However, the clinics were able to dispense these drugs.

Comment #3.19: 232-236 There is an extrapolation of the results that is not supported by the results or any literature at least in this manuscript. Failure to recognise a drug cannot translate to delayed health seeking behaviour. People use antibiotics only when they are sick or for prophylaxis. So if people with unknown HIV status are not using them, will that mean then that they might be HIV positive and therefore delayed health seeking behaviour? Did you make attempts to look at access issues? Like how they got the antibiotics? Were the antibiotics used after getting prescription or just buying on their own? What about the source? Distances to the hospital? Or access to drug stores or pharmacies? I think these would have helped in generating more answers to the questions of your study. Otherwise, use alone cannot be an issue or it can be an issue if we look at reasons for use and how the drugs are accessed.

Author's Response: Thank you for these comments. Unfortunately, these questions are beyond the scope of the study and we would not be able to answer them with the data we have available. However, we agree these are important areas for future research.

Comment #3.20: 252 add 'for' after provided.

Author's Response: Thanks done.

Comment #3.21: 251-261 Is talking about access of cotrimoxazole through primary health care system, which has not been mentioned in this studys data collection or analyses (If it is mentioned and I have missed it, please include a table citation in the discussion). Where is cotrimoxazole use being attributed to healthcare facilities in the results? That is why I mentioned earlier that how they accessed the antibiotics and reasons thereof would have been relevant.

Author's Response: The large majority of health care in Malawi is provided through the primary healthcare system. HIV care in particular, where most co-trimoxazole is provided, is mainly now delivered through the primary healthcare system. We did not capture where participants reported obtaining antibiotics from, so are unable to add data to the table here.

Comment #3.22: 263 Another reason why women were better at recognising the medicines would be their frequent encounter with the medicines as they mostly also take care of children particularly under Five children who are mostly prescribed with antibiotics for almost every disease due to lack of laboratory facilities in most health centres. Flus and colds are also mostly prescribed antibiotics and flus and colds are very common in children.

Author's Response: Yes, thanks we agree. We described this in detail in lines 278-283.

Lines: 278-283:

In Malawi, gender power relations shape household care-seeking practices, with men often presenting at health care facilities critically ill, and having delayed diagnosis of diseases of major public health importance, particularly HIV and tuberculosis.[29] Women spend considerably more time than men seeking care and supporting household members to seek care.[30] These factors are likely to explain why women had better recognition and use of antibiotics.

Comment #3.23: 277-283 This statement is built on no results from this study or no cited literature that this is the case in the areas of the study. This supports my earlier suggestions that reasons for using the antibiotics stated should have been recorded.

Author's Response: Thank you for this observation. By access here we mean in the broader global sense, whereby countries such as Malawi have extremely limited access to second and third-line antibiotics. We agree that future studies might investigate reasons for antibiotic use, but this was not done by the present study.

Malawi-Liverpool-Wellcome Trust Clinical Research Programme
P.O. Box 30096, Chichiri, Blantyre 3
Tel: +2651 1 81 24 23 / +2651 811 918 // Fax: +2651 875 774
